# Exploring chronic disease prevalence in people with intellectual disabilities in primary care settings: A scoping review

**DOI:** 10.1111/jar.12957

**Published:** 2021-11-08

**Authors:** Milou van den Bemd, Maarten Cuypers, Erik W. M. A. Bischoff, Marloes Heutmekers, Bianca Schalk, Geraline L. Leusink

**Affiliations:** ^1^ Radboud Institute for Health Sciences, Department of Primary and Community Care Radboud University Medical Centre Nijmegen The Netherlands

**Keywords:** cardiovascular diseases, chronic disease, chronic obstructive pulmonary disease, diabetes mellitus, intellectual disability, prevalence

## Abstract

**Background:**

Primary care providers require accurate evidence on chronic disease prevalence in people with intellectual disabilities in order to apply this information into practice. This study aimed to map the broadness of literature on chronic disease prevalence in people with and without intellectual disabilities, and to explore main characteristics of these studies.

**Method:**

A scoping review of peer‐reviewed literature was conducted, covering 2000 to February 2020, including literature that discussed chronic disease prevalence in people with and without intellectual disabilities, with similar data collection method for both groups.

**Results:**

Nineteen studies were included. Chronic disease prevalence varied considerably between people with and without intellectual disabilities. Studies differed in their methodologies, country and age groups that were enrolled.

**Conclusions:**

Primary care providers should interpret results on disease prevalence among people with intellectual disabilities in light of the study characteristics. Researchers should always interpret prevalence rates in the context of methodology.

## 1 INTRODUCTION

Unambiguous information on chronic disease prevalence in people with intellectual disabilities is largely lacking (Macrae et al., 2015; Oeseburg et al., 2011). Varying and sometimes even conflicting prevalence rates are presented in the literature (Draheim, 2006; Macrae et al., 2015). Heterogeneity between studies can potentially be reflected in various factors such as sample size, type of data, or methods of identification of intellectual disabilities; making correct understanding and interpretation of chronic disease prevalence in people with intellectual disabilities more complex.

Primary care providers and actors in public health planning require accurate information on chronic disease prevalence to interpret results in terms of chronic diseases being more or less prevalent among people with intellectual disabilities as compared to people without intellectual disabilities (Cooper et al., 2018; Mccarron et al., 2017; Morin et al., 2012; Tyler et al., 2010). Such accurate evidence, that can be applied and translated into practice, is a first necessity in providing optimal healthcare (Lennox et al., 2015). A better insight into the aspects that relate to the inconsistencies in the literature is therefore necessary to help primary care providers and researchers to better understand and accurately interpret prevalence rates of chronic diseases in people with intellectual disabilities.

Chronic diseases such as ischaemic heart disease (IHD), cerebrovascular disease (CVD), diabetes mellitus (DM) and chronic obstructive pulmonary disease (COPD) are among the most common chronic diseases worldwide (Vos et al., 2017). They have the highest impact on both the economic level (Abegunde & Stanciole, 2006; Foster et al., 2006) and patients' individual level, such as their quality of life (Mcknight‐Eily et al., 2009; Sattoe et al., 2014; Shih & Simon, 2008) and risk of mortality (Lauer & Mccallion, 2015; Lozano et al., 2012). The impact of chronic diseases can be even higher for people with intellectual disabilities compared to the general population, as they experience limitations in adaptive behaviour and intellectual functioning (Schalock et al., 2010). As a result, it is more difficult for them to fully comprehend the implications of chronic diseases, and this complicates disease management and results in poorer health outcomes (Van Schrojenstein Lantman‐De & Walsh, 2008).

As chronic diseases are mostly managed in primary care, this setting provides the most complete representation of everyone in the population with and without chronic diseases (Crooks et al., 2015; Harvey et al., 2002). Secondary care settings typically report higher prevalence estimates than primary care settings do, as patients in this setting are more likely to have a chronic illness but may be overrepresented by severe cases (Crooks et al., 2015). It is therefore most relevant to focus on prevalence studies on people with and without intellectual disabilities conducted in primary care settings. Information on the prevalence of diseases such as IHD, CVD, DM and COPD is also used to plan the size and the allocation of healthcare resources (Petrou & Wolstenholme, 2000). Accurate understanding of published prevalence rates is therefore essential. This scoping review therefore aims (1) to map the broadness of published literature on IHD, CVD, DM and COPD prevalence in people with intellectual disabilities compared to people without intellectual disabilities in primary care settings, and (2) to explore main characteristics of these studies.

## 2 METHODS

### 2.1 Study design

This study is a scoping review, a type of review commonly used to map existing literature that ‘exhibits a large, complex or heterogeneous nature’ (Peters et al., 2015, p. 141). They are particularly useful for describing research findings in more detail by taking different research designs into account (Arksey & O'malley, 2005; Munn et al., 2018; Peters et al., 2020). This way, study characteristics that may be deemed important can be mapped and discussed (Munn et al., 2018). This scoping review followed the PRISMA guidelines extension for scoping reviews (PRISMA‐ScR) (Tricco et al., 2018).

### 2.2 Search strategy

To identify eligible studies, the databases of Embase, Medline, PubMed, Web of Science and PsycInfo were electronically searched for publications issued between 1 January 2000 and 7 February 2020. The search strategy was developed in collaboration with a medical research librarian and consisted of a combination of four concepts: intellectual disabilities, prevalence, chronic diseases, and comparison with the general population. Both broad (e.g., ‘chronic diseases’) and specific (e.g., ‘diabetes mellitus’) terms were used in order to ensure that all relevant studies were included in the search results. A complete overview of the search strategy is provided in the Supplement.

### 2.3 Study selection

Studies were included if they:

- were written in the English language;
- reported original data;
- were published in peer‐reviewed journals;
- discussed the prevalence of at least IHD, CVD, DM or COPD;
- addressed the prevalence within (a subgroup of) people with intellectual disabilities compared to people without intellectual disabilities;
- used a data collection method that was identical for people with and without intellectual disabilities.

Studies were excluded if they focused solely on conditions where intellectual disabilities cannot be assumed (i.e., cerebral palsy, autism spectrum disorder); assessed the prevalence of chronic conditions after certain interventions; focused on children only (aged 18 or below); and took place in secondary care settings only (such as hospitals or specialist care).

The initial search was conducted by the first author (MvdB), with the second author (MC) screening a random sample of 10% of all titles and abstracts. Next, the remaining articles were screened full‐text by the first and the second author to assess eligibility. Disagreements were solved by discussion.

### 2.4 Methodological quality assessment

To better judge the results of included studies, all studies deemed eligible for inclusion were evaluated on methodological quality to assess risk of bias. The appraisal tool used – Joanna Briggs Institute Prevalence Critical Appraisal Tool – was created specifically to evaluate studies reporting prevalence data (Munn et al., 2015). The checklist consisted of nine questions and addressed the following issues: sampling, sample size, (non)response rates, description of study participants and country, appropriate statistical analysis, and valid and reliable methods to identify the condition of intellectual disabilities. The first and the second author assessed the studies separately and later reached agreement by discussion.

The results of the quality appraisal checklist were combined into four main topics in order to provide a more structured overview. First, the findings regarding the sample were summarised; this concerned issues such as representativeness, sampling methods and sample sizes. Second, attention was paid to the method of identification of people with intellectual disabilities. Possible influencing factors such as the use of proxy respondents, identification of intellectual disabilities based on formal diagnosis or otherwise, and method of recruiting respondents with intellectual disabilities were taken into account. Third, the manner of identification of chronic diseases was summarised, such as diagnoses in medical records or self‐reported diseases. Last, the type and detail of statistical analyses performed in each study were summarised. For each topic, studies were assessed on a three‐point scale ranging from sufficient (+) to insufficient (−). The assessments are presented in the Supplement.

### 2.5 Data extraction and calculations

All data on relevant chronic diseases were extracted from the included articles. Some studies reported chronic disease prevalence for men and women separately (Mcdermott et al., 2007b) or for age groups separately (Flygare Wallen et al., 2018). In order to achieve comparability, new prevalence rates were calculated by determining the mean of the rates for men and women (not weighted due to unavailability of population size rates) and weighted mean of the rates for the age groups. Thus, one mean prevalence rate for the total study population was computed.

Characteristics of the included studies were described. First, different types of data can be used to report on chronic disease prevalence, such as register or (primary care) medical data. Next, the definition of intellectual disabilities is the way in which intellectual disabilities were operationalised in the included study. Methods for identifying someone as having intellectual disabilities consisted of a medical record of a diagnosis of intellectual disabilities, various screening methods, or information on received services or supports specifically for people with intellectual disabilities (e.g., income support programmes, social services and residential care). Country was defined by the country in which the studies were performed, along with their dominant lifestyle and health policies and their organisation of healthcare. Next, age groups were the ages of the included study groups that were taken into account. Lastly, sample size was the size of the group of people with intellectual disabilities and the comparison group.

## 3 RESULTS

**FIGURE 1:**
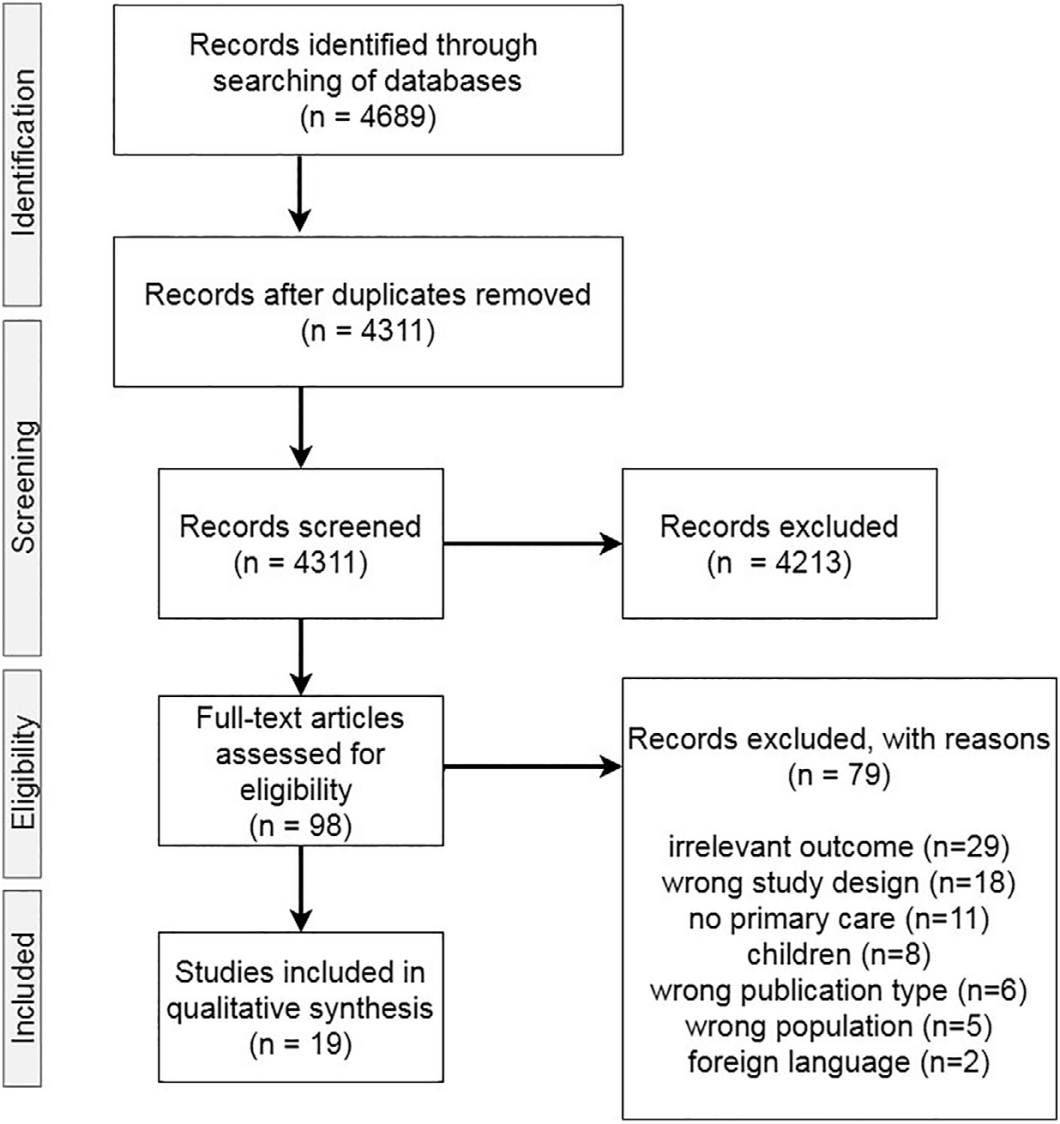
Search results and study selection flow chart, adapted from Moher et al. (2009)

The initial search resulted in 4311 papers, excluding duplicates. After title and abstract screening, 98 articles were assessed full‐text. There was disagreement on 14% of the articles (*n* = 14), on which consensus was reached by discussion. This resulted in 19 studies meeting the inclusion criteria (Figure 1). A complete overview of study characteristics and prevalence rates is shown in Table 1. Country, time period, type of data and characteristics of the study groups are described per study. In this table, prevalence rates in percentages and the odds ratio or other reported calculations are also presented. DM was reported most often (*n* = 18), followed by IHD (*n* = 10), CVD (*n* = 10) and COPD (*n* = 8).

**TABLE 1:** Study and population characteristics of included studies on the prevalence of chronic diseases in people with and without intellectual disabilities

				Characteristics of study groups			Prevalence of chronic diseases in *n* (%)		
Source	Country (time period)	Type of data	Definition of intellectual disabilities as mentioned in study	People with intellectual disabilities	People without intellectual disabilities	Chronic disease	People with intellectual disabilities	People without intellectual disabilities	PR, RR, OR with 95% CI (intellectual disabilities vs. no intellectual disabilities)
Carey et al. (2016)	England (2012)	Primary care database	QOF codes for learning disabilities, no further distinction	18+, registered in primary care; *n* = 14,751	Matched controls; *n* = 86,221	Ischaemic heart disease	244 (1.7)	2316 (2.7)	PR: 0.65 (0.57–0.74)^§^
						Stroke and TIA	267 (1.8)	944 (1.1)	PR: 1.74 (1.52–1.98)^‡^
						DM	1107 (6.9)	3786 (4.4)	PR: 1.64 (1.53–1.75)^‡^
						COPD	160 (1.1)	1184 (1.4)	PR: 0.84 (0.71–0.99)^§^
Cooper et al. (2018)	Area of Greater Glasgow, Scotland (2007–2010)	Primary healthcare register of people with intellectual disabilities	Intellectual disabilities measured by Vineland Scale in levels mild, moderate, severe, profound, and Down syndrome	18+, registered in primary care; *n* = 721	2006/7 QOF data for all adult patients within the area; *n* = 764,672	Coronary heart disease	25 (3.5)	34,711 (4.5)	RR (rate ratio): 0.76 (0.52–1.13)^†^
						Stroke	13 (1.8)	15,008 (2.0)	RR: 0.92 (0.53–1.58)^†^
						DM	46 (6.4)	25,944 (3.4)	RR: 1.88 (1.41–2.51)***
						COPD	9 (1.2)	16,858 (2.2)	RR: 0.57 (0.29–1.09)^†^
Cooper et al. (2015)	Scotland (2007)	Primary care database	A set of Read Codes based on definitions used by NHS Scotland Information Services and from QOF	People with intellectual disabilities (level not reported) aged 18+; *n* = 8014	People without intellectual disabilities aged 18+; *n* = 1,416,364	Coronary heart disease	160 (2.0)	81,307 (5.7)	OR: 0.43 (0.37–0.51)***
						Stroke or TIA	171 (2.1)	36,374 (2.6)	OR: 1.19 (1.02–1.37)^†^
						DM	531 (6.6)	74,300 (5.3)	OR: 1.63 (1.49–1.79)***
						COPD	209 (2.6)	52,898 (3.7)	OR: 0.84 (0.73–0.97)***
Dias et al. (2013)	Correctional centres in Queensland, Australia (2008–2010)	Structured questionnaire in confidential interviews	Screening with Hayes Ability Screening Index (HASI); HASI‐score <85 and attended special school or received diagnosis	Prisoners; *n* = 115	Prisoners, screened with HASI, score >85; *n* = 1164	DM	6 (5.4)	63 (5.1)	aOR: 1.3 (0.5–3.3)^†^
Durbin et al. (2019)	Ontario, Canada (2010)	Health administrative databases	Disabilities income support programmes and algorithm that uses information from diagnostic codes (intellectual disabilities, foetal alcohol syndrome, autism spectrum disorder, other pervasive developmental disorders, chromosomal and autosomal anomalies)	Newcomers aged 19–65; *n* = 2830	Newcomers aged 19–65; *n* = 1,646,803	DM	280 (9.9)	119,768 (7.3)	aPR/RR: 1.97 (1.77–2.20)^§^
						COPD	67 (2.4)	28,343 (1.7)	aPR/RR: 2.11 (1.68–2.66)^§^
Erickson and Kornexl (2016)	Midwestern academic medical centre, USA (2011)	Registration data	ICD‐9 diagnosis of mental retardation or having diagnosis of one of the more common conditions associated with developmental disabilities (autism spectrum disorder, Down syndrome, Williams syndrome, fragile X syndrome, cerebral palsy, foetal alcohol syndrome)	18+, patient in general internal medicine practice; *n* = 183	18+, patient in general internal medicine practice; *n* = 497	Myocardial infarction	3 (1.6)	2 (0.4)^†^	
						Stroke	10 (5.5)	7 (1.4)**	
						DM	19 (10.4)	74 (14.9)^†^	
Erickson et al. (2016)	Midwestern academic medical centre, USA (2012)	Medical records	ICD‐9 diagnosis of condition related to intellectual disabilities: Down syndrome, foetal alcohol syndrome, cerebral palsy, autism spectrum disorder, mental retardation, developmental disabilities, not specified/other	People with intellectual and developmental disabilities aged 40–79 years without a history of cardiovascular disease; *n* = 78	People without intellectual and developmental disabilities aged 40–79 years without a history of cardiovascular disease; *n* = 187	Myocardial infarction (40‐years)	1 (2.3)	0 (0)^†^	
						Stroke (40‐years)	2 (4.7)	0 (0)^†^	
						DM (40‐years)	4 (9.3)	4 (3.1)^†^	
						DM (40–79 years)	9 (11.5)	36 (19.3)^†^	
Flygare Wallen et al. (2018)	Stockholm, Sweden (2010)	Administrative data on healthcare	ICD‐10 diagnosis of moderate, severe, profound, other or unspecified intellectual disabilities, unspecified disorder of psychological development, Down syndrome, trisomy 18, trisomy 13, fragile X syndrome, congenital malformation syndromes, Rett's syndrome, autism spectrum disorder, other childhood disintegrative disorder, Asperger's syndrome, other pervasive developmental disorders	Persons with intellectual disabilities excluding Down syndrome; *n* = 11,785; Persons with Down syndrome; *n* = 1282	Persons without any diagnosis of intellectual disabilities, Down syndrome, or autism spectrum disorder; *n* = 1,996,140	DM (18+, women, intellectual disability vs. no intellectual disability)	251 (8.2)	50,171 (6.3)	OR: 2.40 (2.11–2.73)^‡^
						DM (18+, men, intellectual disability vs. no intellectual disability)	342 (9.0)	64,621 (8.5)	OR: 2.01 (1.80–2.24)^‡^
						DM (18+, women, Down syndrome vs. no intellectual disability)	19 (5.5)	50,171 (6.3)	OR: 1.78 (1.17–2.73)^‡^
						DM (18+, men, Down syndrome vs. no intellectual disability)	15 (3.9)	64,621 (8.5)	OR: 0.70 (0.42–1.17)^†^
Haider et al. (2013)	Victoria, Australia	Survey (general population), administrative database (intellectual disabilities)	Not reported	Proxy respondents on behalf of people with intellectual disabilities; *n* = 897	*n* = 34,168	Stroke	N.R. (2.0)	N.R. (2.5)^†^	
						DM	N.R. (8.9)	N.R. (5.8)*	
Havercamp et al. (2004)	North Carolina, USA (2001)	Survey (general population), registration data and interviews (intellectual disabilities)	Random sample of adults with developmental disabilities receiving special services; self‐reported developmental disabilities	Information obtained via registration/medical data, interviews with person or proxy respondent; *n* = 946	Two groups: No disabilities (*n* = 4358), Disabilities (*n* = 1598)	DM (intellectual disabilities vs. no disabilities)	N.R. (3.9)	N.R. (7.9)	RR: 2.0 (1.4–2.9)*
Hedgeman et al. (2017)	Denmark (1995–2012)	Danish National Patient Registry	Prader‐Willi syndrome, diagnosis made in study period by ICD‐code of DQ871E	All persons with Prader‐Willi syndrome *n* = 155	General population; *n* = 15,500	Myocardial infarction	x	31 (0.2)^§^	
						DM	14 (9.0)	162 (1.0)^§^	
Jansen et al. (2013)	Two Dutch care providers for people with intellectual disabilities aged 50+ (2007)	Medical records of general practice patients in two Dutch care providers and primary healthcare in same region	Indication for residential care and specialist primary healthcare, based on mild, moderate, severe, profound intellectual disabilities, Down syndrome	Individuals aged 50 and over; *n* = 510	All patients aged 50 years and over registered in a large general practice in the same area; *n* = 823	Lifetime prevalence of CVD and/or myocardial infarction	25 (5.7)	36 (4.4)^†^	
Mccarron et al. (2017)	Ireland (2010)	Cohort study (incl. in‐person interviews, questionnaire, and physical health assessment)	Receiving services	Adults aged 50 years or older in intellectual disabilities database; *n* = 478	Adults aged 50 years or older; *n* = 478	Heart attack	7 (1.5)	15 (3.1)^†^	
						DM	52 (11.1)	31 (6.5)*	
Mcdermott et al. (2007b)	Country not reported (1990–2003)	Medical records	ICD‐9 diagnosis of autism spectrum disorder, cerebral palsy with and without mental retardation, psychiatric disabilities with mental retardation, other mental retardation	Adults with intellectual disabilities in primary care medical practices; *n* = 652	Matched patients based on age at entry into general practice; *n* = 1828	DM (total intellectual disabilities group)	61 (9.8)	265 (14.5)	OR: 1.1 (0.8–2.2)^†^
						DM (only mental retardation)	82 (12.7)	265 (14.5)	OR: 1.4 (0.9–2.1)^†^
Mcdermott et al. (2006)	South Carolina, USA (1990–2003)	Medical records	ICD‐9 diagnosis of autism spectrum disorder, cerebral palsy with and without mental retardation, psychiatric disabilities with mental retardation, other mental retardation	Adults with developmental disabilities registered in primary care; *n* = 692	Matched patients based on age at entry into general practice; *n* = 2084	TIA	N.R. (0.3)	N.R. (1.7)^§^	
						DM	N.R. (10.4)	N.R. (15.8)^§^	
						COPD	N.R. (6.4)	N.R. (9.5)^§^	
Mcdermott et al. (2007a)	South Carolina, USA (1990–2003)	Medical records	ICD‐9 diagnosis of autism spectrum disorder, cerebral palsy with and without mental retardation, psychiatric disabilities with mental retardation, other mental retardation	Adults with developmental disabilities registered in primary care; *n* = 692	Matched patients based on age at entry into general practice; *n* = 2084	TIA (women)	N.R. (0.0)	N.R. (1.7)	
						TIA (men)	N.R. (0.5)	N.R. (1.4)	HR: 0.96 (0.64–1.45)^†^
						DM (women)	N.R. (12.3)	N.R. (16.2)	
						DM (men)	N.R. (8.7)	N.R. (13.8)	HR: 1.04 (0.57–1.89)^†^
						COPD (women)	N.R. (4.6)	N.R. (8.6)	
						COPD (men)	N.R. (7.9)	N.R. (10.2)	
Morin et al. (2012)	Quebec, Canada (2010)	Province‐wide mail survey	Receiving services from an agency for intellectual disabiliites and autism spectrum disorder or from social services (eligibility based on AAIIDD definition)	Individuals aged 15 years and older receiving services; *n* = 789	People aged 15 years and older; *n* not reported	DM (intellectual disabilities vs. no intellectual disabilities)	N.R. (8.3)	N.R. (5.1)^†^	
						DM (mild/mode rate intellectual disabilities vs. no intellectual disabilities)	N.R. (8.6)	N.R. (6.4)^†^	
						DM (severe/profound intellectual disabilities vs. no intellectual disabilities)	N.R. (4.8)	N.R. (6.4)^†^	
						DM (Down syndrome vs. no intellectual disabilities)	N.R. (4.2)	N.R. (5.1)^†^	
Perera et al. (2019)	Haringey and London, England (2016–2017)	Health and social care register	Diagnosis of learning disabilities in medical record (from learning disabilities register)	All persons aged 0+ registered in general practice in England; *n* = 1078 (Haringey), *n* = 28,078 (London), *n* = 153,993 (total England)	All persons registered in general practice in England; *n* = 282,478 (Haringey), *n* = 7,559,949 (London), *n* = 33,322,790 (total England)	Coronary heart disease (Haringey (H))	N.R. (0.7)	N.R. (1.6)^§^	
						Coronary heart disease (London (L))	N.R. (0.9)	N.R. (0.0)^§^	
						Coronary heart disease (total England (E))	N.R. (1.1)	N.R. (3.1)^§^	
						Stroke or TIA (H)	N.R. (1.1)	N.R. (0.9)^§^	
						Stroke or TIA (L)	N.R. (1.5)	N.R. (1.1)^§^	
						Stroke or TIA (E)	N.R. (1.7)	N.R. (1.7)^§^	
						DM type 1 (H)	N.R. (0.5)	N.R. (0.2)^§^	
						DM type 1 (L)	N.R. (0.6)	N.R. (0.3)^§^	
						DM type 1 (E)	N.R. (0.7)	N.R. (0.4)^§^	
						COPD	N.R. (1.1)	N.R. (0.9)^§^	
						COPD	N.R. (1.0)	N.R. (1.2)^§^	
						COPD	N.R. (1.1)	N.R. (1.9)^§^	
Tyler et al. (2010)	Cleveland, USA (2005–2008)	Electronic health records	ICD‐9 diagnosis of one of the following: intellectual disabilities, cerebral palsy, chromosomal abnormalities (incl. Down syndrome), pervasive developmental disorders (incl. Autism spectrum disorder), unspecified delay in development, anomalies of the brain	Persons of 18 years or older receiving ongoing healthcare at the Cleveland Clinic; *n* = 1267	One‐to‐one match by age, sex, race and health insurance status with two other patients who received similar care during the same study period; *n* = 2534	Coronary heart disease	33 (3.5)	196 (7.7)	0.43 (0.31–0.60)***
						DM	131 (10.3)	384 (15.2)	0.65 (0.52–0.80)***
						COPD	41 (3.2)	145 (5.7)	0.55 (0.39–0.78)***

*Note*: †, no significant difference; ‡, significant difference, *p*‐level not reported; *p < 0.05; **p < 0.01; ***p < 0.001; §, significance not reported.

Abbreviations: (a)PR, (adjusted) prevalence risk; (a)OR, (adjusted) odds ratio; CI, confidence interval; COPD, chronic obstructive pulmonary disease; CVD, cerebrovascular disease; DM, diabetes mellitus; IHD, ischaemic heart disease; N.R., not reported; RR, relative risk (unless stated otherwise); TIA, transient ischaemic attack; x, size too low to report (1–5 observations).

### 3.1 Characteristics of the included studies

The results of the quality appraisal are depicted in Appendix S1. Eight studies received a high appraisal (++ or +), eight a medium appraisal (±), three a low appraisal (−).

The characteristics of the included studies are described in Table 1. The majority of the included studies (*n* = 14/19) used register or (primary care) medical data to report on chronic disease prevalence, such as medical records or national patient registries. Definition of intellectual disabilities varied across studies, but most based operationalisations on ICD‐9 or ICD‐10 codes (*n* = 9/19). Often, a diagnosis of intellectual disabilities was combined with diagnoses of other conditions, such as autism spectrum disorder (*n* = 8), cerebral palsy (*n* = 6) or foetal alcohol syndrome (*n* = 3). The majority of the studies (*n* = 11/19) identified people with intellectual disabilities through a diagnosis in medical records or through records of services received (*n* = 5/19). Most studies were performed in Western‐Europe (*n* = 8/19). Three studies did not report their country, but were assumed to be performed in the Unites States based on earlier similar work (Erickson et al., 2016; Erickson & Kornexl, 2016; Mcdermott et al., 2007b). In total, seven studies were performed in the Unites States. Most included studies took into account adults aged 18 years or older (*n* = 11/19), others focused on adults aged 40 or 50 years and older or all ages. Lastly, the sample size across studies varied from 78 to 153,993 people with intellectual disabilities, and from 187 to 33,322,790 people without intellectual disabilities.

### 3.2 IHD prevalence

**FIGURE 2:**
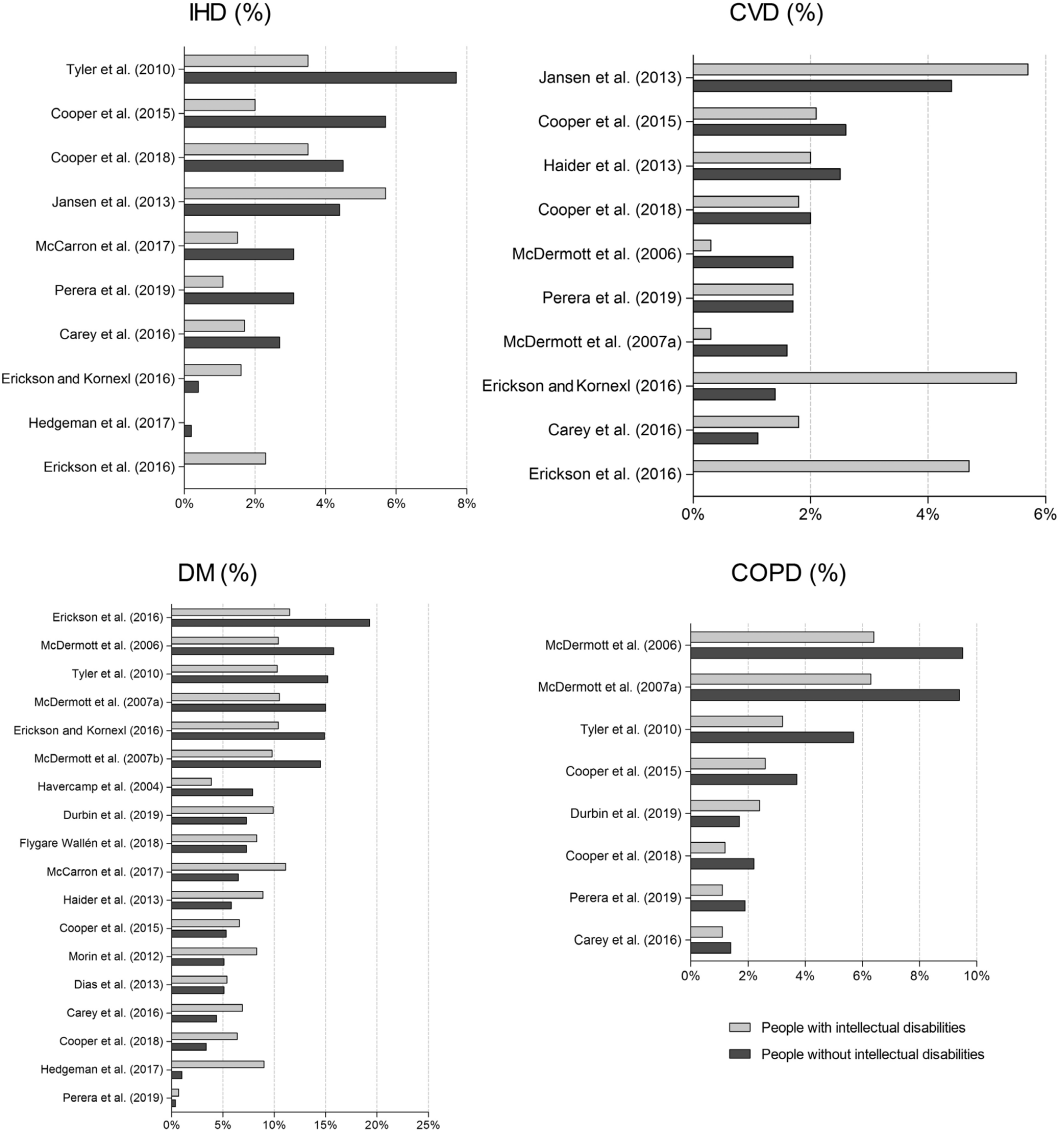
Prevalence of IHD, CVD, DM and COPD (%) in the literature

**FIGURE 3:**
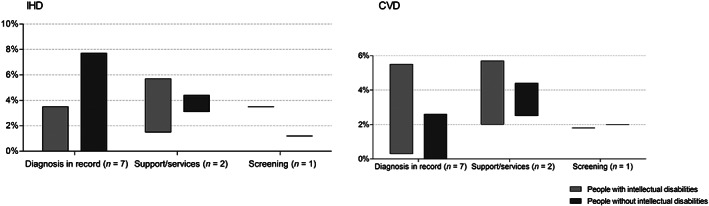
Range in IHD and CVD prevalence (%) in the literature, split by type of identification of intellectual disabilities in data

**FIGURE 4:**
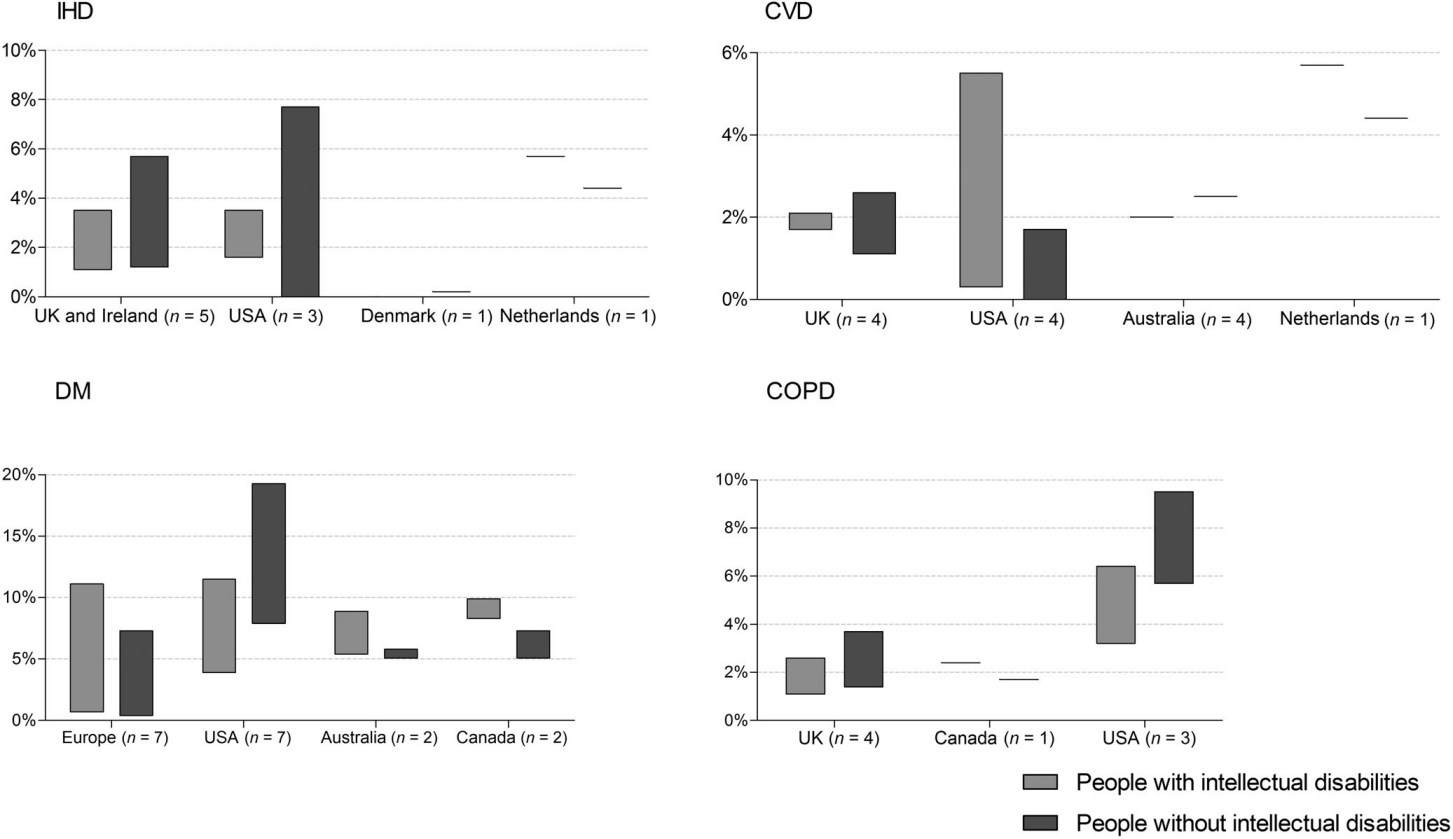
Range in chronic disease prevalence (%) in the literature by country

Studies (*n* = 10/19) reported IHD prevalence rates between 0.0% and 5.7% for people with intellectual disabilities, and 0.0% to 7.7% for people without intellectual disabilities (Figure 2). In most studies, IHD prevalence was lower for people with intellectual disabilities compared to people without intellectual disabilities. One study that stratified by severity levels of intellectual disabilities reported higher IHD prevalence in more severe levels (Jansen et al., 2013). The highest IHD prevalence rates among people with and without intellectual disabilities were found among the studies with a high‐quality appraisal (Jansen et al., 2013; Tyler et al., 2010) (Table 2). The range in IHD prevalence was higher in studies where the population of people with intellectual disabilities was identified through relevant diagnoses in medical records rather than through other methods (Figure 3). The two studies identifying intellectual disabilities through support or services both focused on adults aged 50 years or older (Jansen et al., 2013; Mccarron et al., 2017), of which one shows highest IHD prevalence among people with intellectual disabilities (Jansen et al., 2013). In studies performed in the Unites States, IHD prevalence had the highest range for people without intellectual disabilities compared to other countries (Figure 4). Studies performed in Great Britain utilised larger samples, which likely contributed to lower IHD prevalence compared to other countries.

**TABLE 2:** Summary of patterns in study and population characteristics across prevalence studies

	Ischaemic heart disease	Cerebrovascular disease	Diabetes mellitus	Chronic obstructive pulmonary disease
Quality appraisal	Highest prevalence rates in studies with highest appraisal	No pattern	Highest prevalence rates in general population in studies with negative appraisal	Highest prevalence rates in general population and population with intellectual disabilities in medium appraisal studies
Type of data	No pattern	No pattern	No pattern	No pattern
Definition of intellectual disabilities	No pattern	No pattern	No pattern	No pattern
Method of identification of intellectual disabilities	Higher prevalence in studies using received support/services compared to diagnoses in medical records	Highest prevalence among studies using received support/services compared to other measurements	Highest and lowest prevalence in general population and population with intellectual disabilities among studies using intellectual disabilities‐related diagnoses in medical records	No pattern
Country	In UK and Ireland, higher prevalence in general population compared to population with intellectual disabilities, in the United States other way around	Highest range of prevalence among population with intellectual disabilities in the United States, in UK the smallest	In USA, population with intellectual disabilities has higher prevalence rates compared to general population, in other countries vice versa	Highest prevalence among general population and population with intellectual disabilities in United States, relatively low prevalence in UK and Canada
Age groups	No pattern	Highest prevalence rates in study focusing on elderly (50+ years), lowest among study focusing on younger persons (40− years)	Studies focusing on all ages present lowest prevalence rates in general population and highest prevalence in population with intellectual disabilities	Lowest prevalence in study focusing on all ages
Sample size	No pattern	Most difference in prevalence rates among study using smallest samples	Highest prevalence rates in general population and population with intellectual disabilities in smaller samples, lowest prevalence rates in larger samples	Lower prevalence rates in studies with larger sample sizes, highest prevalence in smallest samples

### 3.3 CVD prevalence

CVD prevalence in the included studies (*n* = 10/19) varied from 0.3% to 5.7% among people with intellectual disabilities, and from 0.0% to 4.4% among people without intellectual disabilities (Figure 2). One study reported prevalence by severity levels: the higher the severity level of intellectual disabilities, the higher the CVD prevalence (Jansen et al., 2013). The range in prevalence among people with intellectual disabilities was higher when diagnoses of intellectual disabilities in medical records were used as the indicator (Figure 3). The United States had the highest range in CVD prevalence among people with intellectual disabilities. In the UK, the range in CVD prevalence was higher among people without intellectual disabilities (Figure 4). The highest CVD prevalence among people both with and without intellectual disabilities was reported by a study including adults aged 50 years and older (Jansen et al., 2013); the lowest prevalence rates were reported by Erickson et al. (2016) who included ages 40 years or less (Table 2). The highest difference in prevalence rates between people with and without intellectual disabilities could be found among the study using the smallest samples (Erickson et al., 2016). Studies performed in Great Britain in general utilised larger samples compared to other countries.

### 3.4 DM prevalence

The prevalence of DM varied in studies (*n* = 18/19) from 0.7% to 11.5% among people with intellectual disabilities, and from 0.4% to 19.3% among people without intellectual disabilities (Figure 2). DM prevalence was mostly higher for people with intellectual disabilities than for people without intellectual disabilities, except in studies that found high prevalence rates among people without intellectual disabilities (>10%). Only two studies distinguished between Type 1 and 2 diabetes (Mcdermott et al., 2006, 2007a). Both the highest and the lowest DM prevalence for people with and without intellectual disabilities were found in studies using diagnoses related to intellectual disabilities in medical records (Table 2). DM prevalence among people with intellectual disabilities was generally lower in the Unites States compared to those without intellectual disabilities, whereas the opposite was true for Western‐Europe (Figure 4). The studies with highest appraisal were performed in Western‐Europe (Cooper et al., 2015; Hedgeman et al., 2017). The two studies focusing on all ages reported the highest prevalence among people with intellectual disabilities and the lowest DM prevalence among people without intellectual disabilities (Hedgeman et al., 2017; Perera et al., 2019). Lastly, the smallest sample size corresponds with the highest DM prevalence in people both with and without intellectual disabilities (Erickson et al., 2016), whereas the lowest prevalence rates can be found in the largest sample size (Perera et al., 2019). The highest DM prevalence among people with and without intellectual disabilities was reported in a study from the Unites States with smallest sample, which focused on the oldest age groups (40–79 years) compared to the other studies (Erickson et al., 2016).

### 3.5 COPD prevalence

Studies on COPD (*n* = 8/19) reported prevalence rates from 1.1% to 6.4% among people with intellectual disabilities, and from 1.4% to 9.5% among people without intellectual disabilities (Figure 2). In all but one study (Durbin et al. (2019), the prevalence of COPD was lower in people with intellectual disabilities compared to people without intellectual disabilities. The highest COPD prevalence was reported by two studies with a medium appraisal (Mcdermott et al., 2006, 2007a). COPD prevalence was highest in the USA compared to studies performed in other countries, and showed the largest differences between people with and without intellectual disabilities (Figure 4). Prevalence rates in the UK were more comparable between people with and without intellectual disabilities, and overall lowest across the included studies. The only study considering all ages reported the lowest COPD prevalence (Perera et al., 2019) (Table 2). A larger sample size was accompanied by a lower COPD prevalence (Perera et al., 2019), a smaller sample size by a higher prevalence (Mcdermott et al., 2006, 2007a).

## 4 DISCUSSION

This scoping review is the first to map the broadness of published literature on chronic disease prevalence in people with intellectual disabilities compared to people without intellectual disabilities. Chronic disease prevalence varied considerably between studies and differed when study characteristics were taken into account. This study builds upon existing chronic disease prevalence reviews by exploring their observations that methodological differences in the included studies could possibly be important in explaining variances in prevalence rates. The reviews mention methodological differences such as operational definition and method of identification of intellectual disabilities, differences in study groups in terms of sex and aetiology of intellectual disabilities, method of data collection, sample size and method of diagnosis of chronic diseases (Jansen et al., 2004; Macrae et al., 2015; Mcvilly et al., 2014; Oeseburg et al., 2011). Other similar reviews either did not take the role of methodological choices into account or focused on different health problems (Fortin et al., 2012; Jansen et al., 2004). This study is therefore the first to offer guidance to primary care providers and researchers in interpreting chronic disease prevalence in people with intellectual disabilities.

This review described characteristics of included studies and identified five valuable aspects that are important when interpreting chronic disease prevalence in people with intellectual disabilities; being type of data, identifying of intellectual disabilities, country, age of the study groups and sample size. These aspects are discussed one by one: First, when interpreting results, one should always be aware of the consequences of different types of data. Studies relying on self‐reported values are at risk of potential bias, which may result in an over‐ or underestimation of a person's ill‐health. In people with intellectual disabilities, self‐reporting can be accompanied by extra challenges (Fujiura & Rrtc Expert Panel on Health Measurement, 2012), and therefore studies often resort to using proxy respondents. However, proxy reporting decreases the validity of the results (Cummins, 2002; Emerson et al., 2013) and complicates comparison between people with and without intellectual disabilities.

Second, this study emphasises the value of recognising the way in which intellectual disabilities are identified and defined across studies. Although most included studies used similar methods for identifying intellectual disabilities (via medical records or records of specific services), chronic disease prevalence was still diverse in these studies. This finding suggests that studies using the same methods for identifying people with intellectual disabilities do not necessarily include the same populations, as people with intellectual disabilities are identifiable via multiple sources. Earlier research supports the finding that using different identification methods as well as different definitions of intellectual disabilities may complicate estimating prevalence rates (Lin et al., 2013).

Only a few countries have national registers from which intellectual disabilities can be identified in a relatively reliable manner; other methods are often less conclusive (Mcconkey et al., 2019). Frequently, many different conditions related to intellectual disabilities were examined simultaneously, but in conditions such as autism or cerebral palsy intellectual disabilities cannot always be assumed (Bryson et al., 2008; Reid et al., 2018).

Third, the country in which studies were performed was relevant for interpreting chronic disease prevalence. Interestingly, in the United States, the prevalence of cardiovascular diseases (IHD and CVD) was consistently higher among people with intellectual disabilities compared to people without intellectual disabilities, whereas COPD and DM in the USA were more prevalent among people without intellectual disabilities. Prevalence of IHD, CVD, DM and COPD was high in the United States among people both with and without intellectual disabilities compared to other countries. A possible explanation is the higher prevalence of unhealthy lifestyles, and consequently obesity levels, in the Unites States (Wang & Beydoun, 2007), given that these diseases are all related to unhealthy lifestyles (Forey et al., 2011; Willett et al., 2006). In addition, some argue that American health promotion policies can be prone to reinforce health inequalities (Goldberg, 2012), whereas European policies seem more inclusive (Fosse, 2011). Furthermore, the differences in primary care systems in the Unites States and European countries can result in different timings in diagnosis and management of chronic diseases (Erler et al., 2011; Mcglynn et al., 2003). When interpreting and comparing health statuses of people with intellectual disabilities residing in the Unites States and Western‐Europe these differences should therefore always be kept in mind.

Fourth, the role of age should always be noted in studies on chronic disease prevalence. Although the life expectancy of people with intellectual disabilities has increased, they often show earlier signs of aging compared with people without intellectual disabilities (Evenhuis et al., 2012), resulting in higher mortality rates (Hosking et al., 2016). Results and comparability between people with and without intellectual disabilities can be affected by this earlier aging effect, as the occurrence of chronic diseases is generally higher with increasing age (Buist et al., 2007; Thomsen & Nordestgaard, 2014), and as several chronic diseases are more common among aging people with intellectual disabilities than among aging people without intellectual disabilities (Krahn et al., 2006). In line with these previous findings, this review found that studies only taking older age groups into account were more likely to report higher prevalence of chronic diseases in people with intellectual disabilities.

Fifth, sample sizes should be critically evaluated when one is interpreting differences in prevalence rates of chronic diseases. In the case of COPD and DM, it could be seen that a higher sample size was accompanied by a lower prevalence, and vice versa. This can be explained by the fact that larger sample sizes are generally better suited to make more precise claims and are more likely to have included a representative sample (Charter, 2003).

### 4.1 Strengths and limitations

This review has some limitations. First, we restricted our scope of chronic disease to IHD, CVD, DM and COPD. Diseases that are more prevalent among people with intellectual disabilities, for instance epilepsy (Mcdermott et al., 2005) or chronic skin disease (Mcdermott et al., 1997), were not taken into account. We chose to focus on the four most prevalent types of chronic conditions that have a large global impact as well as a high impact on the everyday lives of people with intellectual disabilities. Second, few studies included in this review make necessary distinctions, such as between diabetes Type 1 and Type 2, ischaemic or haemorrhagic stroke, or severity levels of intellectual disabilities. However, diabetes Type 1 and Type 2 have different manifestations and aetiology (Zaccardi et al., 2016). Not being able to make these distinctions complicates the formulation of adequate disease management methods for specific diseases.

Notwithstanding these limitations, this review provides the first exploration of literature on chronic disease prevalence rates in people with intellectual disabilities compared to people without intellectual disabilities. Although Jansen et al. conducted a similar review in 2004 (Jansen et al., 2004), they focused solely on the prevalence of several health problems that were not included in this review, such as epilepsy and sensory loss. The current review is in line with another review that explored how methodological choices may influence multimorbidity prevalence rates (Fortin et al., 2012). Comparable to the current review, the authors concluded that type of data, country and age groups are important in assessing multimorbidity in the general population. However, intellectual disabilities were not taken into account (Fortin et al., 2012). This review therefore offers direction in interpreting studies on chronic disease prevalence in people with intellectual disabilities. Second, it offers a first insight into the comparative health regarding chronic diseases of people with intellectual disabilities compared to the general population. Third, a large variety of studies have been taken into account. Although study characteristics such as age or sex are better known influences on prevalence rates (Flygare Wallen et al., 2018; Perera et al., 2019), this review highlights the significance of other, less often examined characteristics, such as type of data. In traditional reviews, the great heterogeneity in study designs, populations and countries is associated with challenges in summarising evidence, but by performing a scoping review it was possible to explore such characteristics in greater depth. Fourth, the fact that we were able to perform a quality assessment increases the legitimacy of the claims made.

### 4.2 Recommendations for future research

This review provides a fruitful basis upon which to build future research on chronic diseases in people with intellectual disabilities. First, as the current review is the first to explore the role of study designs, populations and countries in chronic disease prevalence, this study can be used as a valuable basis for conducting further research, such as a meta‐analysis. In addition, no studies conducted in non‐Western countries were identified. Research demonstrates that chronic diseases represent a high burden in non‐Western, low‐ or middle‐income or less developed countries (Boutayeb & Boutayeb, 2005; Wagner & Brath, 2012). The situation of people with intellectual disabilities is also very different in such countries, but this global difference is not often studied (Emerson et al., 2008). The prevalence rates of IHD, CVD, DM and COPD as presented in this review are therefore a representation of Western countries.

Next to the use of different methods or countries, this review has also identified several important aspects that future research should take into account when both studying and interpreting chronic disease prevalence in people with intellectual disabilities. First, future research should disclose as much as possible the study and population characteristics. Existing guidelines for prevalence studies, such as STROBE or RECORD (Benchimol et al., 2015; Von Elm et al., 2014), are useful tools and should be utilised widely. This way, the need for valid and reliable information on the health of people with intellectual disabilities (Ruddick, 2005) can be better met. Second, in order to make useful claims future studies on chronic disease prevalence should take into account multiple interacting factors, such as age (Erickson et al., 2016; Jansen et al., 2013; Perera et al., 2019) or sex (Flygare Wallen et al., 2018; Mcdermott et al., 2007a), but also factors such as type of data or identification of intellectual disabilities. Additionally, future research should report chronic disease prevalence by severity levels of intellectual disabilities if possible. The few studies that do so report possibly important patterns in chronic diseases (Cooper et al., 2018; Heslop et al., 2019; Jansen et al., 2013). Third, large population studies should be conducted in order to obtain reliable and valid prevalence estimates. In this type of study, entire populations can be taken into account, resulting in thoroughly defined and representative study populations (Lieb, 2013). Because it currently still is difficult to identify people with intellectual disabilities in population datasets (Emerson et al., 2013), future studies should be transparent in the methods used to identify people with intellectual disabilities.

Lastly, comparisons between incidence and prevalence rates can prove interesting research subjects. While prevalence rates are useful for indicating disease burden, incidence rates give insight in the occurrence rate of chronic diseases in populations (Keiding, 1991).

## 5 CONCLUSION

This review adds to the literature by providing a first exploration of the broadness of published literature on chronic disease prevalence in people with intellectual disabilities and by describing main characteristics of these studies. Chronic disease prevalence varies greatly between people with and without intellectual disabilities across studies. Although study characteristics such as country and age group are more apparent influencers in chronic disease prevalence, this review also highlights the importance of other factors that are less often examined, such as type of data and definition of intellectual disabilities. Researchers should therefore acknowledge the influence of study characteristics and methodologies when studying chronic disease prevalence in people with intellectual disabilities. This review underscores the need for transparent and comparable prevalence studies. The great heterogeneity in study characteristics and methodologies complicate generalisation of study results. Rather, this review argues that prevalence rates should always be interpreted in the context of methodology. Only then, primary care providers and public health planners are able to utilise prevalence rates of chronic diseases and apply them into practice.

## CONFLICT OF INTEREST

The authors have declared no conflicts of interest.

## Supporting information


**Supplement**: Supporting Information

## Data Availability

Data available on request from the authors The data that support the findings of this study are available from the corresponding author upon reasonable request.
