# Supplementary material for: Exploring chronic disease prevalence in people with intellectual disabilities in primary care settings: A scoping review
Source: J Appl Res Intellect Disabil. 2021 Nov 8;35(2):382–98. doi: 10.1111/jar.12957 (PMC9298833; doi:10.1111/jar.12957)
Supplement: Supplementary file 1 — Supplement: Supporting Information [file JAR-35-382-s001.docx]

Supplement. Search strategy for PubMed^a^

| Concept | Search terms |
| --- | --- |
| Intellectual disabilities | "Intellectual Disability"[Mesh] OR "Mentally Disabled Persons"[Mesh] OR “Developmental Disabilities/complications”[Mesh] OR “Developmental Disabilities”[Mesh] OR “Specific Learning Disorder”[Mesh] OR intellectual development disorder*[tiab] OR mentally challenged[tiab] OR learning disabilit*[tiab] OR special needs[tiab] OR low IQ[tiab] OR developmental disab*[tiab] OR down syndrome*[tiab] OR downs syndrome*[tiab] OR prader-willi[tiab] OR fragile X[tiab] OR Trisomy 21[tiab] OR Trisomy21[tiab] OR trisomies[tiab] OR mongolism*[tiab] |
| Prevalence | “Prevalence”[Mesh] OR “Epidemiology”[Mesh] OR epidemiology[MeSH Subheading] OR “Cross-sectional studies”[Mesh] OR “Incidence”[Mesh] OR prevalence[tiab] OR epidemiology[tiab] OR incidence[tiab] |
| Chronic diseases | "Chronic Disease"[Mesh] OR "Healthcare Disparities"[Mesh] OR "Health Status"[Mesh] OR "Comorbidity"[Mesh] OR "Morbidity"[Mesh] OR "Multiple Chronic Conditions"[Mesh] OR chronic disease*[tiab] OR chronic illness*[tiab] OR health disparit*[tiab] OR health status*[tiab] OR comorbid*[tiab] OR morbidity[tiab] OR multimorbidity[tiab] OR chronically ill[tiab] OR "Cardiovascular Diseases" [Mesh] OR heart attack*[tiab] OR myocardial infarct*[tiab] OR heart infarct*[tiab] OR Stroke*[tiab] OR cerebrovascular[tiab] OR cva[tiab] OR Coronary artery disease*[tiab] OR coronary arteriosclerosis[tiab] OR atherosclerosis[tiab] OR Peripheral Arterial Disease*[tiab] OR Congenital heart*[tiab] OR heart abnormalit*[tiab] OR heart defect*[tiab] OR Venous thromboembolism[tiab] OR deep vein thrombosis[tiab] OR dvt[tiab] OR pulmonary embolism[tiab] OR lung embolism[tiab] OR pulmonary thromboembolism[tiab] OR lung thromboembolism[tiab] OR "Respiratory Tract Diseases"[Mesh] OR respiratory tract disease*[tiab] OR respiratory hypersensit*[tiab] OR bronchial disease*[tiab] OR Asthma[tiab] OR Asthmas[tiab] OR COPD[tiab] OR Chronic Obstructive Pulmonary Disease[tiab] OR COAD[tiab] OR Chronic Obstructive Airway Disease[tiab] OR Respiratory disease*[tiab] OR "Diabetes mellitus"[Mesh] OR diabetes[tiab] |
| Comparison with general population | “Matched-Pair Analysis”[Mesh] OR “Case-Control Studies”[Mesh] OR “Control Groups”[Mesh] OR “Probability”[Mesh] OR matched pair analys*[tiab] OR case-control stud*[tiab] OR control group*[tiab] OR odds ratio[tiab] OR comparison[tiab] OR compared[tiab] |

^a^ The search terms for Web of Science are similar, but adapted to its specific format.

Supplement. Result of appraisal checklist

|  | **Overall rate** | **Sample** | **Identifying intellectual disabilities** | **Identifying chronic disease** | **Statistical analysis** |
| --- | --- | --- | --- | --- | --- |
| Cooper et al. (2015) | **++** | **+ Sufficient**  Use of register data | **+ Sufficient**  Use of QOF codes for learning disabilities | **+ Sufficient**  Chronic disease(s) identified by diagnoses in medical records | **+ Sufficient**  Methods were described in sufficient detail to properly identify the analytical method. The statistical analysis was sufficient |
| Hedgeman et al. (2017) | **++** | **+ Sufficient**  All people with Prader-Willi syndrome in Denmark are included and matched with general population on 1:100 | **+ Sufficient**  Identification via diagnosis of intellectual disabilities in medical record | **+ Sufficient**  Chronic disease(s) identified by diagnoses in medical records | **+ Sufficient**  Methods were described in sufficient detail to properly identify the analytical method. The statistical analysis was sufficient |
| Carey et al. (2016) | **+** | **+ Sufficient**  Use of register data (primary care database) | **+ Sufficient**  Use of QOF codes for learning disabilities | **+ Sufficient**  Chronic disease(s) identified by diagnoses in medical records | **+/- Debatable**  Methods were described in detail to properly identify the analytical method. Did not report significance, but did report n, %, and PR |
| Durbin et al. (2019) | **+** | **+ Sufficient**  Use of register data, namely health administrative databases | **+/- Debatable**  Identification is algorithm based, but not a validated one | **+ Sufficient**  Chronic disease(s) identified by validated algorithms | **+ Sufficient**  Methods were described in sufficient detail to properly identify the analytical method. The statistical analysis was sufficient |
| Erickson et al. (2016) | **+** | **+ Sufficient**  All available cases are taken into account, but low N in both group with and without intellectual disabilities | **+/- Debatable**  Identification via diagnosis of intellectual disabilities in medical record, but only 1/3 of this population had an official diagnosis | **+ Sufficient**  Chronic disease(s) identified by diagnoses in medical records | **+ Sufficient**  Methods were described in sufficient detail to properly identify the analytical method. The statistical analysis was sufficient |
| Wallen et al. (2018) | **+** | **+ Sufficient**  Administrative data on healthcare, thus use of register data | **+ Sufficient**  Identification via diagnosis of intellectual disabilities in medical record | **+ Sufficient**  Chronic disease(s) identified by diagnoses in medical records | **+/- Debatable**  Methods were described in sufficient detail to properly identify the analytical method. N and % were reported, but confidence intervals and significance levels were not |
| Jansen et al. (2013) | **+** | **+/- Debatable**  Study population consists of residents from two care providers; this is not representative of the larger population | **+ Sufficient**  Institutionalised people with intellectual disabilities are taken into account, thus use of medical records | **+ Sufficient**  Chronic disease(s) identified by diagnoses in medical records | **+ Sufficient**  Methods were described in sufficient detail to properly identify the analytical method. The statistical analysis was sufficient |
| Tyler et al. (2010) | **+** | **+ Sufficient**  Electronic health records and use of register data | **+ Sufficient**  Identification of intellectual disabilities via diagnosis in medical record | **+ Sufficient**  Chronic disease(s) identified by diagnoses in medical records | **+/- Debatable**  Methods were too poorly described to properly identify the analytical method. The statistical analysis was sufficient |
| Cooper et al. (2018) | **+/-** | **- Insufficient**  Use of register data, but underrepresentation of intellectual disabilities (1:1000). Unclear how sampling took place for both groups. Data on age and gender were unknown for general population | **+/- Debatable**  Identification based on screening | **- Insufficient**  Measurement of chronic disease is unclear for the general population | **+ Sufficient**  Methods were described in sufficient detail to properly identify the analytical method. The statistical analysis was sufficient |
| Erickson and Kornexl (2016) | **+/-** | **+/- Debatable**  All available cases are taken into account. A high percentage of people with intellectual disabilities is ‘lost’, unclear whether they do not have CVD risk factors or whether there are no data available | **+/- Debatable**  Identification via diagnosis of intellectual disabilities in medical record, but only 1/3 of ID population had an official diagnosis | **+ Sufficient**  Chronic disease(s) identified by diagnoses in medical records | **+ Sufficient**  Methods were described in sufficient detail to properly identify the analytical method. The statistical analysis was sufficient |
| Haider et al. (2013) | **+/-** | **+ Sufficient**  Sample is randomly selected. There are no differences in non-responders and responders | **- Insufficient**  No definition of intellectual disabilities is given. Only those are included who sought assistance. Proxy respondents were used. People with intellectual disabilities were invited to participate via CATI, but this is not a suitable method for this group | **- Insufficient**  Chronic disease(s) identified by self-reported values | **+/- Debatable**  Methods were described in sufficient detail to properly identify the analytical method. The %, confidence intervals, and significance are reported, but no N. There were few dropouts |
| Mccarron et al. (2017) | **+/-** | **+ Sufficient**  Random representative sample, no significant differences in non-responders and responders | **+/- Debatable**  Identification of intellectual disabilities via service or support, 1/3 of these respondents are proxy respondents | **- Insufficient**  Chronic disease(s) identified by self-reported values | **+ Sufficient**  Methods were described in sufficient detail to properly identify the analytical method. The statistical analysis was sufficient |
| Mcdermott et al. (2006) | **+/-** | **+/- Debatable**  Use of medical records, unknown whether it is a random sample | **+ Sufficient**  Identification of intellectual disabilities via diagnosis in medical record | **+ Sufficient**  Chronic disease(s) identified by diagnoses in medical records | **+/- Debatable**  Methods were described in sufficient detail to properly identify the analytical method. Only % was reported, no confidence intervals or significance levels |
| Mcdermott et al. (2007b) | **+/-** | **+/- Debatable**  Use of medical records, unknown whether it is a random sample | **+/- Debatable**  Identification of intellectual disabilities via diagnosis in medical record | **+ Sufficient**  Chronic disease(s) identified by diagnoses in medical records | **+ Sufficient**  Methods were described in sufficient detail to properly identify the analytical method. The statistical analysis was sufficient |
| Mcdermott et al. (2007a) | **+/-** | **+/- Debatable**  Medical records, but unclear whether it concerns a random sample or whether sample size is adequate; the matching performed was lower than 1:2 | **+ Sufficient**  Identification of intellectual disabilities via diagnosis in medical record | **+ Sufficient**  Chronic disease(s) identified by diagnoses in medical records | **+/- Debatable**  Methods were described in sufficient detail to properly identify the analytical method. The statistical analysis was appropriate, but no N reported. Men with and without ID were not statistically compared, although that information was available |
| Perera et al. (2019) | **+/-** | **+/- Debatable**  Use of register data, but no information on descriptive characteristics of populations | **+ Sufficient**  Identification of intellectual disabilities via diagnosis in medical record | **+ Sufficient**  Chronic disease(s) identified by diagnoses in medical records | **+/- Debatable**  Methods were described in sufficient detail to properly identify the analytical method. The statistical analysis was limited: only % was reported, no N, confidence intervals, or significance levels. |
| Dias et al. (2013) | **-** | **+/- Debatable**  Adequacy of sample size is uncertain as there was no power calculation. There is no information on non-response, but response rate seems low | **+/- Debatable**  Identification based on screening | **- Insufficient**  Chronic disease(s) identified by self-reported values | **+/- Debatable**  Methods were described in sufficient detail to properly identify the analytical method. The significance level was not reported |
| Havercamp et al. (2004) | **-** | **+/- Debatable**  Two different sources for groups with and without intellectual disabilities . Sample size of second group is higher than that of the first group. No information on non-response | **+/- Debatable**  Use of three sources to identify people with intellectual disabilities, use of proxy respondents to obtain information on this group. | **- Insufficient**  Chronic disease(s) identified by self-reported values | **+/- Debatable**  Methods were poorly described. The statistical analysis was sufficient, but the group of people with intellectual disabilities sometimes had low response |
| Morin et al. (2012) | **-** | **+/- Debatable**  Random sample, but no information on non-response or response rate within study | **+/- Debatable**  Only 7.5% of surveys were filled in by people with intellectual disabilities themselves (rest proxy). Those with intellectual disabilities not receiving any services were not included | **- Insufficient**  Chronic disease(s) identified by self-reported values | **+/- Debatable**  Methods were described in sufficient detail to properly identify the analytical method. The statistical analysis was limited |
